# The Dental Engine

**Published:** 1888-05

**Authors:** 


### THE DENTAL ENGINE.

While the dental appliances of the day have in some respects simplified practice, in others they have complicated it. It is a mistake to suppose that a piece of machinery can be made to supply a lack of skill on the part of the operator. It may make practicable an operation that was impracticable before its invention, but it requires a greater amount of skill and dexterity, for there is the demand for the added knowledge required to run the machine. All the many appliances of the dentistry of to-day are but embarrassments, unless the operator knows how to employ them to the best advantage. There is little doubt that the dental engine has been the cause of the loss of many teeth; not through any fault in the machine, but because of improper and unskillful use. The dentist who has been accustomed to work with a hand-drill, for instance, when he takes in hand the engine, is not prepared for the rapidity of its action, and before he knows it he has sacrificed tissue that should have been saved.

It is a fact, too, that when an operator has, for a time, been working with dull burs and changes them, unless he uses the most extreme care he will, if working in soft dentine, do mischief before he is aware of it. After all, there is nothing like the old-fashioned excavator for conscientious work. No cavity should be wholly prepared by means of the engine bur. The rapidly revolving point conveys no sensation to the brain of the dentist. With it he cannot feel the exact line between sound and unsound structure. The necessary tremulousness of a point in rapid revolution destroys all delicacy of touch.

Necrosed bone is distinguished from sound by its peculiarly gritty and grating sensation beneath a metallic point. But it is impossible accurately to detect this with a revolving bur. So the softened dentine within a cavity of decay cannot be detected with the engine. The bur is excellent for cutting out decayed tissue, but it should always be directed by a probe which has explored the way before it. The excavator should be substituted for the bur at every step, and with it the diseased tooth-bone should be clearly defined. This being cut out, the engine should stop until a further examination has been made. In this manner the danger of cutting away too much is avoided, and the work is intelligently done.

Let any dentist who is accustomed to do all his excavating with the engine go carefully over all the surfaces of a cavity that is supposed to be ready for the introduction of the filling, and he will often be very much surprised at finding softened dentine where he had supposed it all removed, and he will perhaps have made clear to his apprehension the cause for certain hitherto inexplicable failures. Use the engine, by all means, but not without the aid of the exploratory excavator.

